# Differences in Assessing Loneliness Among Japanese Older Adults: A Comparison of Family Physicians and Nurses

**DOI:** 10.3390/jcm15062255

**Published:** 2026-03-16

**Authors:** Kazutaka Yoshida, Aya Goto, Ichiro Kawachi

**Affiliations:** 1 Department of Social Medicine, Graduate School of Medicine, Hirosaki University, Hirosaki 036-8562, Japan; 2 Institute of Global Well-being Science, Hirosaki University, Hirosaki 036-8562, Japan; 3 Department of Global Health and Population, Harvard T.H. Chan School of Public Health, Boston, MA 02115, USA; agoto@hsph.harvard.edu; 4 Center for Integrated Sciences and Humanities, Fukushima Medical University, Fukushima 960-1295, Japan; 5 Department of Social and Behavioral Sciences, Harvard T.H. Chan School of Public Health, Boston, MA 02215, USA; ikawachi@hsph.harvard.edu

**Keywords:** loneliness, family physicians, nurses, primary health care

## Abstract

**Background/Objectives**: Loneliness is highly prevalent in Japan and has become a major public health concern. Although primary health care professionals are often the first to encounter lonely patients, loneliness is subjective and difficult to detect in routine clinical practice. This study aims to examine how family physicians and nurses assess patient loneliness, and whether their approaches differ. **Methods**: This mixed-methods study comprised two surveys administered in Japanese family medicine clinics. Survey 1 (August 2020) was a cross-sectional questionnaire involving patients aged ≥ 50 years (*n* = 470), six family physicians, and seven nurses, of whom one responded on behalf of the group. Patient loneliness was measured using the UCLA (University of California, Los Angeles) Loneliness Scale (Version 3) and served as the reference standard. Physicians and nurses independently assessed patient loneliness based on medical records. Sensitivity, specificity, and predictive values were calculated. Survey 2 (August–September 2023) used an open-ended questionnaire completed by the same physicians and nurses, with responses analyzed using quantitative text mining to explore assessment perspectives. **Results**: Based on the UCLA scale, 38% of patients were classified as lonely. Compared to each other, family physicians demonstrated a higher sensitivity (45.3%) but lower specificity (67.4%), whereas nurses showed a lower sensitivity (21.8%) but higher specificity (84.5%). Text mining revealed that family physicians emphasized relational quality and psychological context, sometimes identifying loneliness even when there is no apparent lack of social connections. In contrast, nurses tended to define loneliness in terms of clearly observable social circumstances and emphasized patients’ subjective acknowledgment. **Conclusions**: Family physicians and nurses employ distinct yet complementary approaches to identifying loneliness in primary health care. Collaborative, role-based strategies may enhance the accurate detection of loneliness and support more effective patient care.

## 1. Introduction

Japan has a high prevalence of loneliness in the population [1]. In 2021, the Japanese government appointed a Minister of State for Loneliness and Isolation within the Cabinet Office, becoming the second country after the United Kingdom (UK) [2,3]. Japan has conducted national surveys on loneliness three times, revealing that 40% of respondents report feeling lonely—similar to findings in the UK [2,3,4]. This percentage is comparable to the United States, where nearly half of individuals report experiencing loneliness [5,6,7]. The U.S. Surgeon General has also recently highlighted loneliness as an epidemic [7]. Given the rapid population aging in these countries, especially in Japan, loneliness among older adults has increasingly been recognized as a major public health concern [3]. National surveys and policy documents in Japan identify older adults as a population at elevated risk for loneliness (30% of adults aged 65 years and older) and social isolation (more than 20% of adults aged 80 years and older report insufficient communication), and similar priorities are seen in the UK [2,3,4]. On the other hand, chronic loneliness persisting over several years is not more prevalent in Japan, the UK, or the U.S. than in other countries [8]. Although loneliness is a universal emotion, cultural factors partly shape its experience. Cultural differences can influence not only loneliness but also social isolation [9]. As a result, comparing prevalence rates across countries can be challenging.

Loneliness significantly impacts mental health and cognitive function, while social isolation is closely linked to declines in physical health and increased mortality [10,11]. When loneliness leads to social disconnection, it increases the risk of cardiovascular disease and premature death [12]. The World Health Organization recommends screening for loneliness in healthcare settings, as it represents an important risk factor for future morbidity and mortality [12]. Loneliness is a subjective state characterized by distress arising from a perceived gap between desired and actual social experiences [7,10]. In contrast, social isolation is an objective condition marked by limited social relationships, roles, group memberships, and interactions. Some individuals may not be socially isolated but still experience loneliness [7,10]. Recognition of loneliness is, therefore, crucial in primary health care [13]. However, in the clinical setting, patients rarely seek medical care for loneliness per se [14]. Family physicians trained in primary health care and nurses specializing in patient care may need to take a more active role in diagnosing and addressing loneliness [15].

Unlike objective social isolation, loneliness is subjective and often invisible, making it challenging to identify [16]. Family physicians and nurses frequently struggle to recognize loneliness in Japan due to time constraints. Family physicians may handle as many as 18 appointments within 3 h, allocating only 5–10 min per patient [17,18,19,20]. While pressed for time, they play a vital role in understanding patients’ lives beyond surface-level complaints, delving into their living conditions and family backgrounds while managing routine and urgent cases [21,22,23,24]. Similarly, nurses juggle multiple responsibilities, such as conducting health checks, assessing living conditions, and reporting findings to physicians, leaving limited time to address emotional needs [25]. Nurses, who often maintain detailed records of patients’ lifestyles, may refer patients to specialists when necessary. However, these records are not designed for comprehensive diagnostic purposes, further complicating efforts to address loneliness in busy clinical settings [26].

Although validated loneliness scales with established cut-off values are widely used in research [27,28,29], their routine use in primary health care may be limited because the time required for administration does not always fit within standard clinical workflows [18,20]. Consequently, family physicians and nurses frequently rely on informal, experience-based assessments. However, little empirical research has examined how different primary health care professionals identify loneliness or whether their assessment tendencies differ. Clarifying these processes is essential for developing feasible, role-based strategies for identifying loneliness in everyday clinical practice.

Therefore, research is currently needed on the criteria used by medical professionals to identify patient loneliness in primary health care settings, particularly in comparing family physicians and nurses. This study aims to explore the differences between Japanese family physicians and nurses in assessing and detecting patient loneliness.

## 2. Methods

The study was conducted in two phases. Survey 1, administered in August 2020, included patients (of the 532 individuals approached, 492 responded, and 470 were included in the analysis), six family physicians, and seven nurses, with one nurse responding as a representative. Survey 2, conducted in August 2023, involved three of the six family physicians and seven nurses who participated in Survey 1. The purpose of Survey 1 was to evaluate the sensitivity, specificity, positive predictive value (PPV), and negative predictive value (NPV) of physician and nurse assessments of patient loneliness. Survey 2 employed text-mining analysis of responses to open-ended questions to explore why physicians and nurses identified loneliness among their patients.

### 2.1. Survey 1:

#### 2.1.1. Study Design and Participants

The study was cross-sectional and employed a self-administered questionnaire. It was conducted at two family medicine clinics affiliated with the Department of Community and Family Medicine (from April 2024, the Department of General Internal Medicine and Family Medicine), Faculty of Medicine, Fukushima Medical University. The clinics employed family physicians certified by the Japanese Primary Care Association. Patients aged 50 or older who attended regular clinic visits between 1 August and 31 August 2020, were eligible for inclusion. Patients were excluded if they were attending their first visit, were assessed by family physicians or nurses as unable to understand the study or complete the questionnaire, or received palliative or end-of-life care. Data collection also included six family physicians and seven nurses who treated these patients, provided they agreed to participate in the study, with one nurse responding as a representative of the group.

#### 2.1.2. Questionnaire Format and Administration

Questionnaires were paper-based and self-administered. The patient questionnaire consisted of 33 items printed on both sides of a single sheet and required approximately 10–15 min to complete. The family physician and nurse questionnaires each consisted of a single item and required only a few minutes to complete. All participants completed the questionnaires by hand using a pen.

#### 2.1.3. Survey Items

Patients self-assessed their loneliness using the UCLA (University of California, Los Angeles) Loneliness Scale (3rd edition) [27] before or after consultation. The scale scores ranged from 20 to 80, with a score of 44 or higher indicating loneliness. A Japanese version of this questionnaire has been developed and has shown adequate reliability and validity [28,29]. This version consists of twenty questions, evaluated by a total score. Nine of the questions are reverse-scored, and all questions have four possible responses: “never,” “rarely,” “sometimes,” and “always.” A different method was used to assess family physicians’ and nurses’ perceptions of patient loneliness. Before the consultation, family physicians and nurses reviewed the patient’s medical records from the previous six months. They then answered the question, “Based on your review of this patient’s medical records, do you believe that he or she is likely to be experiencing loneliness?” using a four-point scale: “yes,” “possible,” “unlikely,” or “no.”

Additional patient data included basic characteristics (age, gender, education, employment, and marital status), living conditions and social networks (housing, living conditions, and community activities), lifestyle behaviors (smoking and drinking), current medical conditions (hypertension, dyslipidemia, diabetes mellitus, cerebrovascular disease, cardiovascular disease, and depression), and medical services (period of clinic visit, long-term care insurance, and physical disability certificate). For basic characteristics, age was categorized with a cutoff at 65 years, and education was classified into two groups: completion of junior high school or high school and above. Living conditions and social network indicators included whether the patient resided in a nursing home or other institution, cohabiting with others, and participating in community or neighborhood-led organized activities. Current medical conditions were self-reported by the patients. For medical services, the period of hospital visits was categorized with a cutoff of 5 years, which is commonly regarded as reflecting sustained patient–physician relationships and stable longitudinal care [30].

#### 2.1.4. Survey Procedure and Participant Flow

Data were collected using three separate questionnaires for patients, family physicians, and nurses, each linked by a pre-assigned identification number. At both sites, family physicians and nurses independently completed their questionnaires based on medical record documentation from the preceding six months. Physicians completed the questionnaire immediately before the consultation, and nurses completed theirs before the pre-examination assessment. Importantly, clinicians did not have access to patients’ questionnaire responses. At Site 1 (with an appointment system), identification numbers were assigned in advance according to the appointment order. After the consultation, physicians explained the study and obtained written informed consent. Patients then completed the questionnaire either immediately after the visit or during waiting time, depending on clinical workflow. For walk-in patients, identification numbers were assigned sequentially and procedures were conducted in the same manner. At Site 2 (without an appointment system), eligible patients were approached in the outpatient reception or waiting area by physicians, nurses, or administrative staff. After obtaining written informed consent, patients received a numbered questionnaire and a sealed envelope. Patients completed the questionnaire during waiting time and returned it in a sealed envelope to prevent clinicians from viewing the responses. All questionnaires were collected separately at designated collection points to maintain independence of responses.

#### 2.1.5. Analysis

Patients were categorized into four groups based on family physicians’ and nurses’ assessments of loneliness versus the patients’ own self-assessment. Family physicians’ and nurses’ ratings of patients were grouped into two categories: “assessed as lonely” (responses of “yes” or “possible”) and “not assessed as lonely” (responses of “unlikely” or “no”). Similarly, patients’ self-assessment of loneliness was categorized as “lonely” (UCLA Loneliness Scale score of 44 or above) and “not lonely” (score below 44). Using patients’ self-assessments as the gold standard, we calculated sensitivity, specificity, PPV, NPV, and the Kappa coefficient for assessments made by family physicians. The same analyses were performed for assessments made by nurses. Additionally, the Kappa coefficient was calculated to assess agreement between family physicians’ and nurses’ evaluations. All analyses used Stata/SE version 18.0 (StataCorp LLC, College Station, TX, USA).

### 2.2. Survey 2:

#### 2.2.1. Study Design and Participants

This was a self-administered survey with open-ended questions designed for quantitative text analysis. In August and September 2023, the family physicians and nurses who participated in Survey 1 were invited to complete an online open-ended written questionnaire. The survey consisted of a single question: “When do patients feel lonely?” The average time required to complete the questionnaire was approximately 15–20 min. Responses were provided in Japanese, and the analysis was conducted in Japanese. Survey 2 was exploratory in nature and aimed to complement the quantitative findings of Survey 1. All clinicians who had participated in Survey 1 were invited, and those who consented were included in the analysis. Because the purpose was to identify general linguistic patterns rather than to generate in-depth qualitative theory, achieving formal data saturation was not the primary methodological objective.

#### 2.2.2. Analysis

Text mining analyses were performed using KH Coder, version 3. The study included ten participants (three family physicians and seven nurses), with a total of 65 sentences analyzed. The analysis was conducted on a sentence-by-sentence basis. Frequently used words were identified, and a subgraph analysis of the co-occurrence network was conducted to classify these words into significant topics. The Jaccard coefficients were calculated to measure the edge strength in the co-occurrence network, and “random walks” were applied for subgraph detection [31]. The diagram displayed the 60 strongest associations, with closely related words color-coded to indicate their groupings.

## 3. Results

### 3.1. Survey 1:

#### 3.1.1. Patient Characteristics

A total of 532 individuals were surveyed, with 492 (92%) responding. After excluding home care patients (*n* = 12) and patients with dementia (*n* = 14), 470 respondents were included in the final analysis. The basic characteristics of the patients are summarized in Table 1. Regarding the survey sites, 56.4% of participants were recruited from Site 1. The mean age of respondents was 70.1 years (standard deviation [SD] = 9.42), with 51.9% being male. Among participants, 73.7% had completed high school or higher education, 47.2% were employed, and 29.4% were unmarried, divorced, or bereaved. Most participants (98.1%) lived in their own homes, 12.9% lived alone, and 50.9% participated in community or neighborhood-led organized activities. Additionally, 73.5% were non-smokers, and 49.1% were non-drinkers. Hypertension was the most common medical condition, affecting 62.1% of participants, while other conditions were less prevalent. Most (64.3%) had been regular patients for over five years. Furthermore, 94.3% did not have long-term care insurance, and 84.5% did not possess a physical disability certificate.

#### 3.1.2. Family Physicians’ and Nurses’ Ratings of Patient Loneliness

Table 2 and Table 3 present the discrepancy between patients’ self-assessments of loneliness on the UCLA Loneliness Scale and family physicians’/nurses’ ratings of patient loneliness. Among the 470 respondents, 179 (38%) were classified as lonely based on the UCLA Loneliness Scale. For patients identified as lonely by the scale, the sensitivity, specificity, PPV, and NPV of family physicians’ assessments were 45.3%, 67.4%, 46.0%, and 66.7%, respectively. For nurses’ assessments, the sensitivity was 21.8%, the specificity was 84.5%, the PPV was 46.4%, and the NPV was 63.7%. Cohen’s Kappa coefficient, indicating agreement, was 0.13 (*p* = 0.003) between patient self-assessments and family physicians’ perceptions and 0.07 (*p* = 0.04) between patient self-assessments and nurses’ perceptions. The Kappa coefficient between family physicians’ and nurses’ perceptions was 0.27 (*p* < 0.001).

### 3.2. Survey 2:

#### 3.2.1. Frequently Used Words by Family Physicians and Nurses

The ten participants (three family physicians and seven nurses) wrote 65 sentences in total, explaining the reasons why they rated their patients as lonely. Data could not be collected from three of the six family physicians who participated in Survey 1 due to illness or because they did not consent to the survey.

The top three frequently used words by family physicians (limited to nouns and verbs, excluding proper nouns, adjectives, and adverbs) were:

1. Family (家族), relationship (関係) (7 occurrences each),
2. Society (社会) (6 occurrences),
3. Patient (患者) (4 occurrences).

Similarly, the top four frequently used words by nurses (limited to the same as above) were:

1. Person (人) (17 occurrences),
2. Family (家族) (11 occurrences),
3. Society (社会) (7 occurrences),
4. Patient (患者) (6 occurrences).

Among the frequently used words, loneliness (孤独), feel (感じる), think (思う), consider (考える), myself (自分), and themselves (本人) were excluded from the enumeration above because they commonly appear in question-and-answer formats and lack distinctiveness in interpretation.

#### 3.2.2. Subgraph Analysis of Co-Occurrence Networks by Family Physicians and Nurses

**Figure 1:**
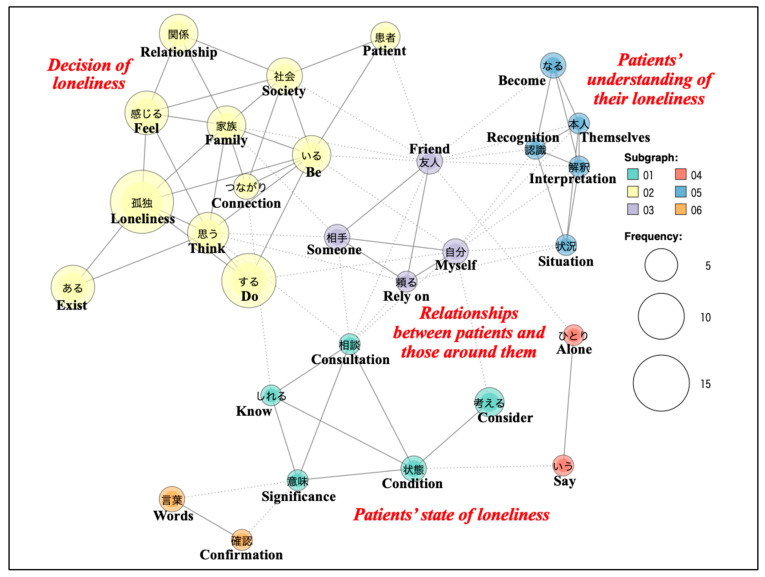
Subgraph analysis of a co-occurrence network among the words used by the family physicians.

For family physicians, the subgraph analysis of a co-occurrence network (Figure 1) based on words used two or more times identified four significant topics, including ‘Decision of loneliness’ (yellow), ‘Patients’ understanding of their loneliness’ (blue), ‘Relationships between patients and those around them’ (purple), and ‘Patients’ state of loneliness’ (green). Close relationships between words were shown with solid lines, while slight relationships were represented with dotted lines. Color-coded subgraphs were created for words connected by solid lines. The size of the circles corresponded to the frequency of word occurrence. Regarding the “Decision of loneliness,” one family physician stated, “I often encounter patients who feel lonely even when they have family.” Another family physician stated, “I think it is possible to feel lonely even in good relationships, perhaps because people hold back around others.” These comments indicate that family physicians identified loneliness in some patients who did not appear socially isolated. Regarding the “Patients’ understanding of their loneliness,” one family physician stated, “Even if a patient does not interpret (recognize) their situation as loneliness, a family physician may still assess it as loneliness.” This suggests that family physicians’ assessments were not based solely on patients’ self-reports.

**Figure 2:**
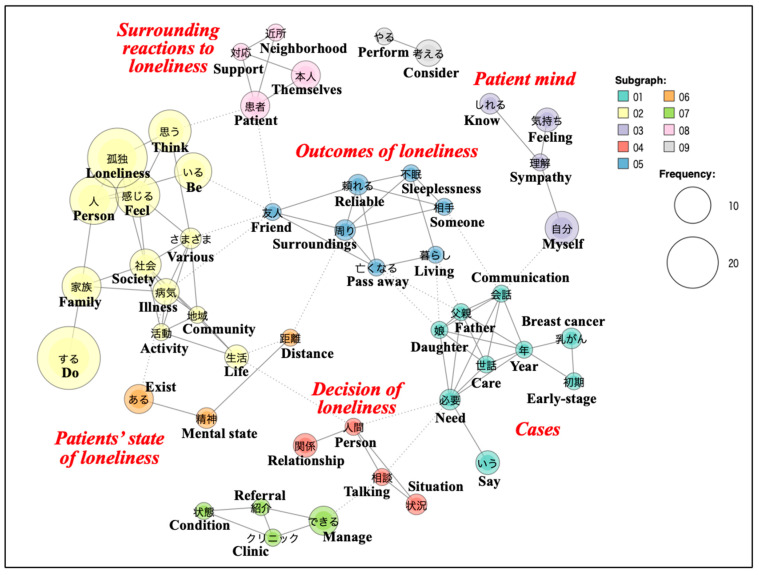
Subgraph analysis of a co-occurrence network among the words used by the nurses.

For nurses, the subgraph analysis of a co-occurrence network (Figure 2) among words used two or more times showed six significant topics, including “Surrounding reactions to loneliness” (pink), “Patient mind” (purple), “Outcomes of loneliness” (blue), “Patients’ state of loneliness” (yellow), “Decision of loneliness” (red), and “Cases” (green). Regarding the “Patients’ state of loneliness,” one nurse stated, “Loneliness occurs when there are no family members or relatives who can be contacted, met with regularly, or spoken to, and when there is no one to consult when help is needed.” Another nurse stated, “It arises when connections to society, the community, and family diminish, leading to reduced conversation and activity levels and increased vulnerability to illness.” These statements describe loneliness in terms of specific social conditions. Regarding the “Surrounding reactions to loneliness,” one nurse stated, “Some individuals in the same situation might not feel lonely. I believe whether someone feels lonely is determined by their patient’s perception.” This reflects an emphasis on patients’ subjective experiences in assessing loneliness. Regarding the “Outcomes of loneliness,” one nurse stated, “For elderly individuals whose spouse has passed away, who have no friends nearby, whose children have their own families and do not live close by, and whose only support is their son, there are no neighborhood connections and sibling relationships are poor.” This statement illustrates how nurses described loneliness based on combinations of observable social circumstances.

## 4. Discussion

This study is the first to explore the differences in how Japanese family physicians and nurses identify patient loneliness within primary health care practice settings. It examines the accuracy and subgroup texts that enable family physicians and nurses to assess loneliness in their patients.

Family physicians assessed a higher proportion of patients who experienced loneliness than nurses, indicating a relatively higher sensitivity and, therefore, fewer missed cases (false negatives) in Survey 1. However, because their specificity was lower than that of nurses, family physicians may have misclassified more patients without loneliness as lonely (false positives). This pattern is consistent with the general tendency in primary health care, in which brief screening methods emphasize sensitivity over specificity [32]. Conversely, nurses demonstrated a higher specificity, suggesting a relative strength in minimizing false positives. However, their notably low sensitivity indicates a greater likelihood of overlooking patients who were actually experiencing loneliness (false negatives). This trend resembles findings from a meta-analysis conducted in hospital and nursing home settings, which showed a similar pattern in nurses’ ability to detect depressive symptoms among patients [33]. Despite these differences, the PPVs of family physicians and nurses were nearly equivalent, indicating that, when a patient was assessed as “lonely,” there was approximately a 46% probability that the patient was indeed experiencing loneliness. Because the prevalence of loneliness in this study was relatively high (38%), as measured by the UCLA Loneliness Scale (Version 3), differences in specificity between physicians (67%) and nurses (85%) had little impact on PPV [34,35]. Nurses’ higher specificity resulted in a slightly higher PPV, whereas their lower sensitivity contributed to a somewhat lower NPV compared with family physicians, who produced fewer false negatives [36]. Taken together, these findings suggest that family physicians tend to “cast a wider net,” whereas nurses tend to “narrow the focus,” and that these complementary assessment characteristics may be best applied in accordance with clinical roles, objectives, evaluation criteria, and care environments.

Survey 2 revealed that family physicians adopted an evaluative approach that broadly identified patients’ loneliness. Their narratives included statements such as “some patients feel lonely even with family” and “patients may be assessed as lonely even if they themselves do not indicate it as such.” These findings suggest that family physicians do not assess loneliness solely on the basis of patients’ self-reports or observable social circumstances; rather, they emphasize the quality of relationships and the psychological context. This perspective is consistent with prior research conceptualizing loneliness as a subjective psychological experience arising from a discrepancy between desired and actual social relationships [37,38] and with studies highlighting the importance of not overlooking potential loneliness in primary health care settings [13,16]. By including patients who do not appear socially isolated in their assessments, family physicians may be less likely to underestimate loneliness.

In contrast, nurses tended to assess loneliness only when multiple specific and observable conditions were present, such as the absence of family or confidants, reduced social interaction, and insufficient support for daily living. They also placed clear emphasis on patients’ subjective perceptions, adopting the stance that “if the individual does not feel lonely, we do not judge them as lonely.” This approach can be interpreted as reflecting a high-specificity evaluative strategy that is less prone to misclassifying non-lonely individuals as lonely. This assessment framework aligns with theoretical perspectives that conceptualize loneliness as a subjective experience, while also being consistent with arguments emphasizing the importance of assessment reproducibility and the avoidance of overdiagnosis in clinical practice and public health interventions [39,40]. Furthermore, studies on loneliness scales and screening have suggested that restricting evaluation criteria may reduce false positives but may do so at the expense of sensitivity [34,41]. Taken together, the assessment tendencies demonstrated by nurses in this study appear to reflect a pragmatic approach in which loneliness is recognized only when clearly defined conditions are met, rather than through a broadly inclusive assessment strategy.

It is essential to explore how family physicians and nurses can collaborate effectively to address loneliness in patients within the limited time available in clinical practice. This proposed collaborative approach aligns with the mobilization of the healthcare sector, one of the national strategies recommended by the U.S. Surgeon General to promote social connection [7]. Our study suggests a two-stage consultation model could be beneficial: the family physician conducts an initial screening for loneliness, followed by a more detailed conversation with the nurse. In the UK, a social prescribing initiative has been implemented where physicians screen patients for loneliness and refer them to link workers, who provide comprehensive listening and support [42]. Similarly, a model project in Japan involves collaboration between physicians, nurses, and other health professionals to assess patients for loneliness and social isolation, offering in-depth listening and support [43]. By recognizing the strengths of family physicians and nurses and clearly defining their respective roles, these initiatives can be further strengthened and more effectively address the needs of patients. It may be helpful to illustrate how such complementary roles could function in routine practice. For example, during a standard consultation, the family physician may briefly screen for possible loneliness based on relational or contextual cues, even when patients do not explicitly report feeling lonely. When potential loneliness is suspected, the patient could then be referred to the nurse for a more focused and structured assessment, including confirmation of subjective feelings by the UCLA Loneliness Scale and evaluation of social support conditions. Notably, a shorter 3-item version of the scale is also available [44]. Patients identified as clearly experiencing loneliness could subsequently receive extended listening support, care coordination, or linkage to community resources. In this way, physicians may “cast a wider net,” while nurses help refine and confirm cases, allowing efficient use of limited consultation time while minimizing both missed cases and over-identification.

Our findings do not suggest that loneliness should be medicalized or treated in the same manner as psychiatric disorders. Rather, they highlight the importance of recognizing loneliness as a clinically relevant condition that shapes patients’ health trajectories and care needs. By elucidating differences in how family physicians and nurses assess loneliness, this study contributes to a practical understanding of how loneliness may be identified in real-world primary health care settings without relying exclusively on standardized screening tools. This study has several limitations. First, the data for this study were collected only from two family medicine clinics in Fukushima Prefecture, Japan, and therefore cannot be generalized. However, it is worth mentioning as data from one clinical site. Second, Survey 2 did not yield a sufficient amount of text. Therefore, it is not possible to determine whether theoretical saturation has been reached.

## 5. Conclusions

This study shows that family physicians and nurses differ in how they identify patient loneliness in primary care. Family physicians tend to prioritize sensitivity, identifying a broader range of potentially lonely patients, whereas nurses emphasize specificity, limiting judgments to clearly defined cases. These complementary assessment patterns suggest that collaborative, role-based approaches improve the accurate identification of loneliness and support more effective care delivery in primary health care settings.

## Figures and Tables

**Table 1:** Basic characteristics of participants and UCLA Loneliness Scale (Version 3) (*n* = 470).

				UCLA Loneliness Scale (Version 3)		
				*n* (%)		
		*n*	%	Loneliness (≥44)	No Loneliness (<44)	*p*-Value ^a^
Site	Site 1	265	56.4	109 (60.9)	156 (53.6)	0.122
	Site 2	205	43.6	70 (39.1)	135 (46.4)	
Basic characteristics						
Age (years)	≥65	342	72.8	122 (68.2)	220 (75.6)	0.078
	<65	128	27.2	57 (31.8)	71 (24.4)	
Gender	Male	244	51.9	98 (54.8)	146 (50.2)	0.335
	Female	226	48.1	81 (45.2)	145 (49.8)	
Education	Junior high school and below	123	26.3	56 (31.6)	67 (23.1)	0.042
	High school or above	344	73.7	121 (68.4)	223 (76.9)	
Employment	Employee	218	47.2	80 (45.5)	138 (48.3)	0.559
	Non-employee	244	52.8	96 (54.5)	148 (51.7)	
Marital status	Married	326	70.6	107 (60.8)	219 (76.6)	<0.001
	Unmarried, divorced or bereaved	136	29.4	69 (39.2)	67 (23.4)	
Living conditions and social network						
Housing	Your own home	457	98.1	171 (96.6)	286 (99.0)	0.073
	Nursing homes and other institutions	9	1.9	6 (3.4)	3 (1.0)	
Living conditions	Cohabiting	405	87.1	145 (81.5)	260 (90.6)	0.004
	Living alone	60	12.9	33 (18.5)	27 (9.4)	
Community activities ^†^	Participating	235	50.9	71 (40.6)	164 (57.1)	0.001
	Not participating	227	49.1	104 (59.4)	123 (42.9)	
Lifestyle behaviors						
Smoking	No	344	73.5	122 (68.5)	222 (76.6)	0.154
	Yes	64	13.7	28 (15.7)	36 (12.4)	
	Former smoker	60	12.8	28 (15.7)	32 (11.0)	
Drinking	No	227	49.1	95 (54.3)	132 (46.0)	0.224
	Sometimes	123	26.6	42 (24.0)	81 (28.2)	
	Every day	112	24.2	38 (21.7)	74 (25.8)	
Current medical conditions						
Hypertension	Yes	292	62.1	75 (41.9)	103 (35.4)	0.158
	No	178	37.9	104 (58.1)	188 (64.6)	
Dyslipidemia	Yes	168	35.8	120 (67.0)	181 (62.4)	0.310
	No	301	64.2	59 (33.0)	109 (37.6)	
Diabetes mellites	Yes	99	21.1	139 (77.6)	232 (79.7)	0.593
	No	371	78.9	40 (22.4)	59 (20.3)	
Cerebrovascular disease	Yes	16	3.4	172 (96.1)	282 (96.9)	0.635
	No	454	96.6	7 (3.9)	9 (3.1)	
Cardiovascular disease	Yes	45	9.6	159 (88.8)	266 (91.4)	0.356
	No	425	90.4	20 (11.2)	25 (8.6)	
Depression	Yes	17	3.6	171 (95.5)	282 (96.9)	0.438
	No	453	96.4	8 (4.5)	9 (3.1)	
Medical services						
Period of clinic visit (years)	<5	167	35.7	70 (39.3)	97 (33.5)	0.198
	≥5	301	64.3	108 (60.7)	193 (66.5)	
Long-term care insurance	Using	25	5.7	13 (7.9)	12 (4.4)	0.125
	Not using	414	94.3	152 (92.1)	262 (95.6)	
Physical disability certificate	Using	18	15.5	7 (16.3)	11 (15.1)	0.862
	Not using	98	84.5	36 (83.7)	62 (84.9)	

^†^ Community activities: Activities within the community or neighborhood-led organized activities, etc. ^a^ Chi-square test was adopted for the analysis of categorical variables.

**Table 2:** Distribution of family physicians’ ratings of patient loneliness, along with sensitivity, specificity, PPV, and NPV.

		UCLA Loneliness Scale (Version 3)		Total	* Kappa Coefficient	*p*-Value	Sensitivity	Specificity	^†^ PPV	^††^ NPV
		Loneliness (≥44)	No Loneliness (<44)							
		179	291	470						
		(38%)	(62%)	(100%)						
Family physician assessment of patient loneliness	Yes	81 (17.2%)	95 (20.2%)		0.13	0.003	45.3%	67.4%	46.0%	66.7%
	No	98 (20.9%)	196 (41.7%)							
Discrepancy between the family physician and the nurse					0.27	<0.001				

* Landis and Koch: 0.00–0.20 Slight, 0.21–0.40 Fair, 0.41–0.60 Moderate, 0.61–0.80 Substantial, 0.81–1.00 Almost perfect. ^†^ PPV, positive predictive value. ^††^ NPV, negative predictive value.

**Table 3:** Distribution of family nurses’ ratings of patient loneliness, along with sensitivity, specificity, PPV, and NPV.

		UCLA Loneliness Scale (Version 3)		Total	* Kappa Coefficient	*p*-Value	Sensitivity	Specificity	^†^ PPV	^††^ NPV
		Loneliness (≥44)	No Loneliness (<44)							
		179	291	470						
		(38%)	(62%)	(100%)						
Nurse assessment of patient loneliness	Yes	39 (8.3%)	45 (9.6%)		0.07	0.04	21.8%	84.5%	46.4%	63.7%
	No	140 (29.8%)	246 (52.3%)							
Discrepancy between the family physician and the nurse					0.27	<0.001				

* Landis and Koch: 0.00–0.20 Slight, 0.21–0.40 Fair, 0.41–0.60 Moderate, 0.61–0.80 Substantial, 0.81–1.00 Almost perfect. ^†^ PPV, positive predictive value. ^††^ NPV, negative predictive value.

## Data Availability

The original contributions presented in this study are included in the article. Further inquiries can be directed to the corresponding author.
